# Evaluation of the clinical feasibility of cone-beam computed tomography guided online adaption for simulation-free palliative radiotherapy

**DOI:** 10.1016/j.phro.2023.100490

**Published:** 2023-08-31

**Authors:** Alannah Kejda, Alexandra Quinn, Shelley Wong, Toby Lowe, Isabelle Fent, Maegan Gargett, Stephanie Roderick, Kylie Grimberg, Sarah Bergamin, Thomas Eade, Jeremy Booth

**Affiliations:** a Northern Sydney Cancer Centre, Royal North Shore Hospital, Sydney, NSW, Australia; b University of Sydney, Sydney, Australia; c Sydney Medical School, University of Sydney, Sydney, NSW, Australia

**Keywords:** Simulation-free radiotherapy, Online adaption, Radiotherapy, Palliative

## Abstract

**Background and purpose:**

Simulation-free radiotherapy, where diagnostic imaging is used for treatment planning, improves accessibility of radiotherapy for eligible palliative patients. Combining this pathway with online adaptive radiotherapy (oART) may improve accuracy of treatment, expanding the number of eligible patients. This study evaluated the adaptive process duration, plan dose volume histogram (DVH) metrics and geometric accuracy of a commercial cone-beam computed tomography (CBCT)-guided oART system for simulation-free, palliative radiotherapy.

**Materials and methods:**

Ten previously treated palliative cases were used to compare system-generated contours against clinician contours in a test environment with Dice Similarity Coefficient (DSC). Twenty simulation-free palliative patients were treated clinically using CBCT-guided oART. Analysis of oART clinical treatment data included; evaluation of the geometric accuracy of system-generated synthetic CT relative to session CBCT anatomy using a Likert scale, comparison of adaptive plan dose distributions to unadapted, using DVH metrics and recording the duration of key steps in the oART workflow.

**Results:**

Auto-generated contours achieved a DSC of higher than 0.85, excluding the stomach which was attributed to CBCT image quality issues. Synthetic CT was locally aligned to CBCT anatomy for approximately 80% of fractions, with the remaining suboptimal yet clinically acceptable. Adaptive plans achieved a median CTV V95% of 99.5%, compared to 95.6% for unadapted. The median overall oART process duration was found to be 13.2 mins, with contour editing being the most time-intensive adaptive step.

**Conclusions:**

The CBCT-guided oART system utilising a simulation-free planning approach was found to be sufficiently accurate for clinical implementation, this may further streamline and improve care for palliative patients.

## 1 Introduction

Palliation of symptoms resulting from oncological disease make up approximately 40–70% of radiotherapy courses internationally [1], with a primary aim of delivering rapid improvement in patient quality of life. Accelerated treatment pathways have been implemented to facilitate timely radiotherapy intervention [2], often using simplified planning and treatment techniques [3], [4], [5]. More recently, utilisation of stereotactic body radiotherapy and dose escalation in palliation of oligometastasis has increased [6], [7], [8], requiring sophisticated planning modalities, such as intensity modulated radiotherapy (IMRT) to achieve precision dose deposition.

Simulation-free radiotherapy is an accelerated pathway where radiotherapy planning is performed on diagnostic computed tomography (dCT) datasets with on-couch treatment imaging [9], [10], [11]. The pathway can enable same-day consultation and treatment, which may extend access to a new group of palliative patients. In practice, patient position and target anatomy in diagnostic scans may not be reproducible at treatment, adding uncertainty to treatment delivery particularly when sophisticated plans are delivered [9], [11], [12]. Integrating simulation-free radiotherapy with online adaptive radiotherapy (oART) where fast contouring and replanning are performed on an image-of-the-day, could realise the full potential of the pathway by accounting for these changes.

Feasibility of oART to the pelvic region has been examined for radical radiotherapy patients, with both Magnetic Resonance (MR) imaging-guided [13], [14], [15], and cone-beam computed tomography (CBCT) -guided systems [16], [17], [18] reporting that adaptive plan was preferred approximately 95% of the time. CBCT-guided oART was able to be delivered in approximately 17.5 mins, while MR-guided oART took an average of 45 mins, both modalities found contouring edits to be the most time-consuming process. Further work is required to determine if online adaption is feasible for simulation-free, palliative patients.

Mittauer et al utilised the dCT to generate a reference palliative plan, before using magnetic resonance imaging (MRI)-guided oART to deliver the plan-of-the-day based on actual treatment geometry [19]. This workflow employed a 3D conformal planning technique, rather than IMRT, to ensure efficiency. Nelisson et al investigated the use of dCT IMRT planning along with a CBCT-guided oART system [20]. The paper outlined preliminary investigations demonstrating the feasibility of a dCT oART pathway in a retrospective review. Early outcomes from the FAST-METS trial for single fraction treatment of bone metastases, were reported utilising the same technique [21], where the average time between patient consult and delivery of oART was 85 mins. Further investigations with a broader patient cohort (soft tissue targets and primary disease), varied fractionation schedules and simultaneous integrated boost (SIB) are still required.

The purpose of this study was to evaluate the feasibility of CBCT-guided online adaption for a broader patient cohort, varied fractionations, and SIB for palliative patients. We determine if the system can deliver complex dose distributions while accounting for geometric variation and dosimetric uncertainty typical of the simulation-free cohort. This work also aims to expand on existing literature and includes our clinical experience and palliative case mix and to discuss the advantages and limitations of this emerging technique.

## 2 Materials and Methods

Evaluation of a CBCT-guided oART system for simulation-free palliation was undertaken using patient datasets in an offline setting and patients treated prospectively with online adaption, see Table S1. The offline cohort comprised of 10 palliative patients previously treated in our department with a conventional unadapted workflow, totalling 29 fractions. Their simulated planning dataset, structure set and CBCTs from clinical treatment were used in a test software environment that emulated the oART contour generation process offline.

The patient cohort treated with online adaption consisted of 20 palliative patients treated with CBCT-guided oART (2 patients had multiple treatment sites), totalling 44 fractions. Each patient reference plan used IMRT with a beam arrangement that minimised integral dose, optimised using dose volume histogram (DVH) goals ranked by importance. 18 of 23 prospectively treated sites were planned with a SIB to clinical target volume (CTV) or gross tumour volume (GTV). The patient position in the dCT was reproduced for treatment delivery. Patients from both cohorts provided consent for their anonymised data to be used for study and publication (local ethics committee reference LNR/15/HAWKE/355).

To be eligible for the simulation-free pathway, dCTs met the following criteria: i) treatment volume extent visualised, ii) relevant patient outline present, iii) patient position able to be reproduced on treatment and iv) dataset extended a minimum of 5 cm beyond the planning target volume (PTV).

**Fig. 1:**
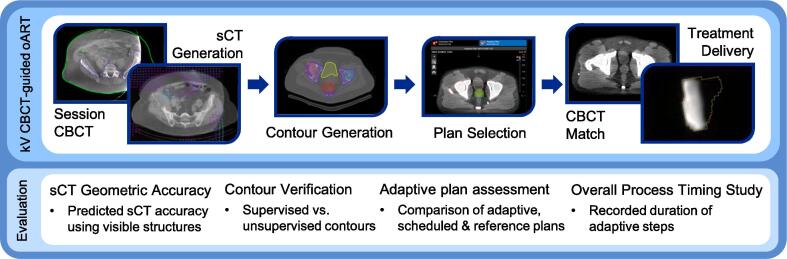
Brief outline of the CBCT-guided oART workflow and evaluation items included in this study.

The CBCT-guided oART system (Ethos™ versions 1.0–1.1; Varian Medical Systems, Palo Alto, CA) and associated workflow has been described in previous publications [16], [20], [21], [22], employing the Acuros External Beam calculation algorithm (AXB) algorithm. A simplified overview of this workflow and tests conducted is outlined in Fig. 1. Feasibility of simulation-free palliative radiotherapy was assessed for each step of the workflow, including the generation of synthetic CT, contours, and adaptive plans, along with the duration of each step.

### 2.1 Synthetic CT geometric accuracy

The CBCT-guided oART system generated the synthetic CT for each fraction via deformable image registration (DIR) of the reference image (the planning dCT) to the session CBCT. Adaptive plan optimisation and dose calculation was completed on the synthetic CT, rather than the session CBCT. During CBCT-guided oART, ‘body’ and ‘bones’ contours were generated by HU threshold-based, auto-segmentation of the synthetic CT and were visualised together with the session CBCT during adaption. The user could not visualise or adjust the synthetic CT online, therefore these contours were used as surrogates of synthetic CT geometric agreement with CBCT anatomy.

The alignment of ‘body’ and ‘bones’ contours to session CBCT anatomy for each fraction of the online adapted patient cohort was assessed by a single expert observer using custom qualitative visual scoring tool derived from the AAPM TG-132 registration uncertainty assessment tool [23], defined in Table S2. The Likert scale assigned a score of 1 for perfect alignment of contours and CBCT in the volume of interest (VOI), while an unacceptable alignment would result in a score of 5. The VOI was defined as volume including a 1 cm expansion of the PTV and any regions of beam entry. Repeated scores of 5 assigned to the generated synthetic CT would suggest the oART system is not suitable for a simulation-free approach.

### 2.2 Contour verification

Contour generation was evaluated utilising offline patient data. The oART system auto-generated contours (unsupervised contours), were compared to clinician edited and approved contours (supervised contours), where the latter accurately represented CBCT anatomy and were considered ground truth. Contour comparison was completed using Dice Similarity Coefficient (DSC). The following contours were evaluated, targets, stomach, liver, kidneys, small bowel, bladder and rectum. The median DSC for each contour was evaluated per patient before the median of the cohort was calculated. The DSCs of stomach contours were stratified per the quality of CBCT dataset, where poor quality CBCTs were defined as containing gas or artefacts within 1 cm of the stomach. DSC scores of <0.7 for multiple organs would limit the feasibility of the oART system for simulation-free applications due to reliance on clinician supervision.

### 2.3 Adaptive plan assessment

The quality of adaptive dose distributions created by the CBCT-guided oART system were assessed for the patient cohort treated with online adaption. The adaptive plan accounted for session CBCT anatomy by reoptimising the reference plan with predefined DVH goals, and calculated on the synthetic CT. The ‘scheduled’ (unadapted) plan was the reference plan recalculated on the synthetic CT. Both adaptive and scheduled plans were compared to the reference plan calculated on the dCT using DVH metrics, where the median DVH metric was evaluated per patient before the median of the cohort was calculated.

CTV and PTV V95% were evaluated with the primary objective defined as CTV V95% ≥ 95%. Organs-At-Risk (OARs) were evaluated and included oesophagus, spinal canal, small bowel, bladder and rectum. Clinical goals for OARs were D0.2 cm^3^ < 10 Gy or 30 Gy, for single fraction or fractionated plans, respectively, with the exception of fractionated small bowel with D0.2 cm^3^ < 25 Gy. For use of the oART system to be feasible, the adaptive plans must reproduce reference metrics and outperform the scheduled for a majority of fractions.

### 2.4 Timing study

**Fig. 2:**
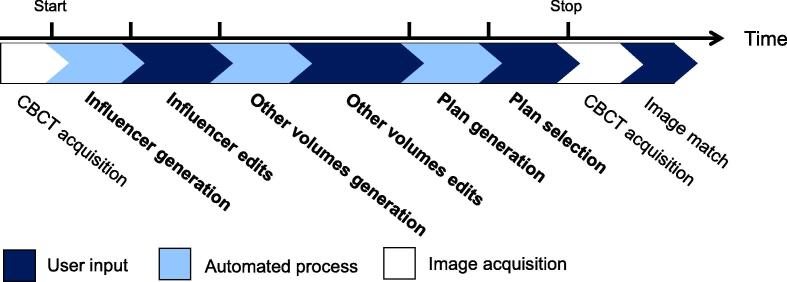
Step-by-step diagram of the kV CBCT-guided oART workflow broken into specific tasks, where the ticks indicate specific timepoints recorded. The duration of the following tasks; influencer generation, influencer edits, other volumes generation and edits, plan generation, and plan review and selection were evaluated in the timing study.

A timing study was conducted with the cohort treated with online adaption to characterise the duration of oART processes. The time of CBCT acquisition was recorded, as well as after the completion of each step to measure task duration of each fraction. The adaptive tasks assessed can be seen in Fig. 2, identified in bold. The system generated certain anatomic contours named influencers, aiding target propagation through structure-guided DIR. All other contours were generated later. Processes not unique to online adaption, such patient setup and treatment delivery, were not assessed. Total adaptive process duration of <20 mins would be necessary for feasible simulation-free palliation.

## 3 Results

### 3.1 Synthetic CT accuracy

Fig. S1 shows the alignment score of ‘body’ and ‘bones’ contours compared to CBCT, where the ‘body’ contour agreed within 2 mm of the session CBCT patient outline (score of 1) for 85% of fractions. The remainder agreed within 2–5 mm (score of 2) suggesting the synthetic CT patient outline reproducibly represented the CBCT.

The ‘bones’ contour aligned to the session CBCT bone edges to within 5 mm (scores of 1 and 2) for 77% of fractions. However, disparity in performance of the remaining fractions was observed, with misalignment of between 10 and 20 mm detected. No 'body’ or ‘bones’ contours were assigned a score of 5 (alignment unacceptable); thus no fractions were aborted.

### 3.2 Contour generation verification

**Fig. 3:**
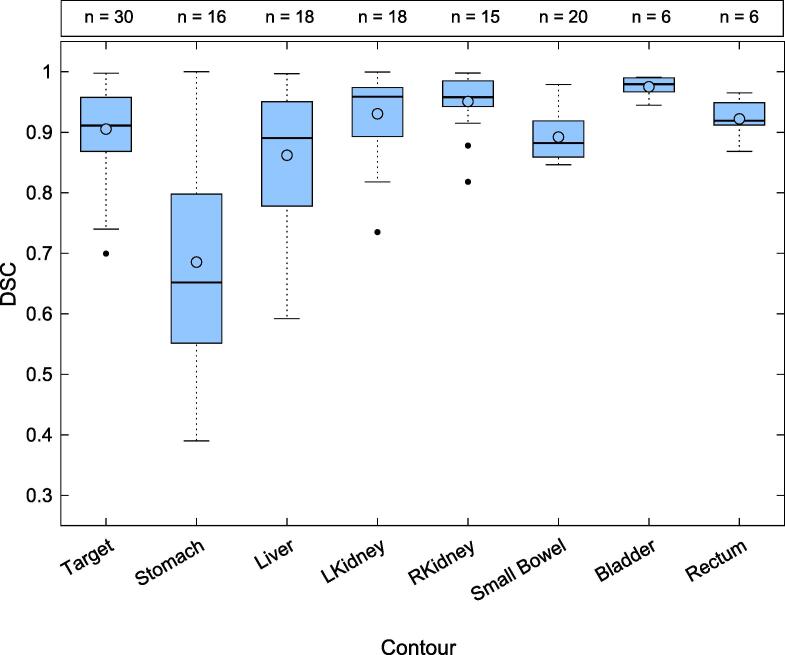
Comparison of auto-generated (unsupervised) to clinician segmented (supervised) contours using dice similarity coefficient score. The number of fractions evaluated for each contour is reported above the graph. The line denotes the median, and the circle denotes the mean DSC of each contour-type.

The median DSC comparing unsupervised to supervised contours for targets, kidneys, bladder and rectum was >0.90, shown in Fig. 3. Liver and small bowel structures reported a median DSC of 0.88 and 0.86, respectively. Sub optimal performance was observed for the stomach contours with a median DSC < 0.61. The median stomach DSC stratified to poor (n = 6) and good quality (n = 10) images were 0.56 (0.39–0.74) and 0.86 (0.54–0.99), respectively.

### 3.3 Adaptive plan assessment

**Fig. 4:**
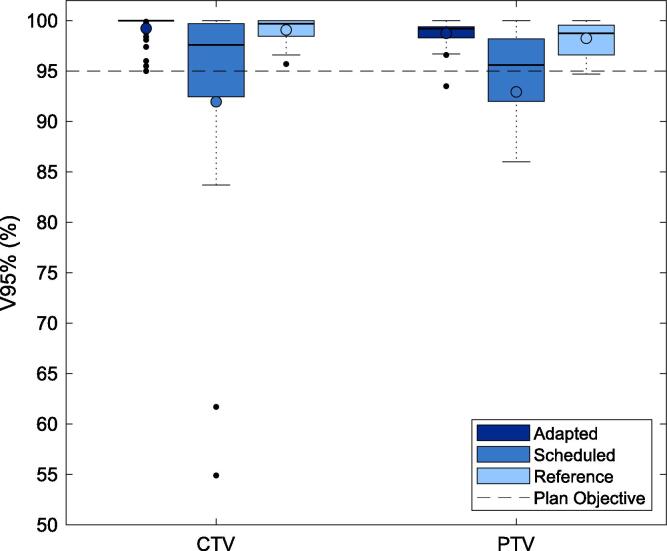
Comparison of CTV and PTV V95% metrics for adapted and scheduled plans of each fraction of oART, evaluated using session synthetic CT and anatomy. The reference plan for each treatment site evaluated on the dCT was also compared. A minimum coverage of CTV V95%>95% represented the primary palliative plan metric. The line denotes the median, and the circle denotes the mean metric performance.

Adapted plans consistently achieved higher CTV and PTV dose coverage than scheduled plans, shown in Fig. 4. The adapted median CTV and PTV V95% was 99.5% and 99.2% respectively, meeting the primary palliative plan objective (CTV V95% ≥95%) in all cases. The median CTV and PTV V95% metrics for scheduled plans were both found to be 95.6%, with 15 out of the 44 fractions failing the primary plan objective.

**Fig. 5:**
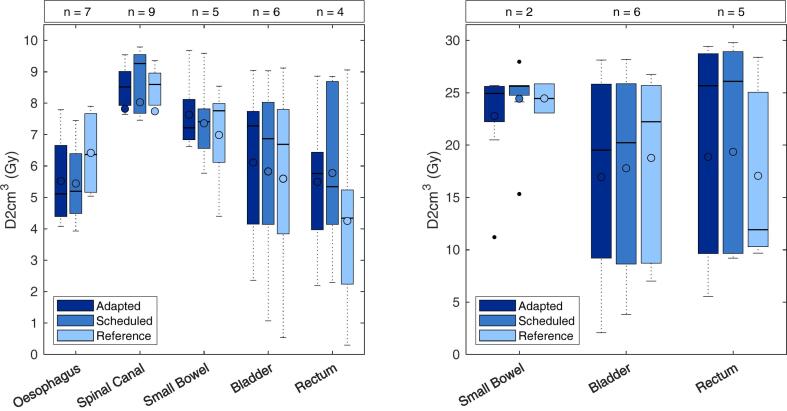
Comparison of OAR D2cm^3^ metric for a) single fraction treatment and b) fractionated treatment for adapted and scheduled plans of each fraction of oART, evaluated using session synthetic CT and anatomy. The reference plan for each treatment site evaluated on the dCT was also compared. The line denotes the median, and circle denotes the mean metric performance.

The median D0.2 cm^3^ of adapted plans were comparable to scheduled and reference plans for the OARs assessed, shown in Fig. 5a and b. Only the small bowel D0.2 cm^3^ < 25 Gy was violated, and in that instance the objective was not met in the reference plan. No adaptive or scheduled plans violated other OAR clinical goals.

### 3.4 Timing study

**Fig. 6:**
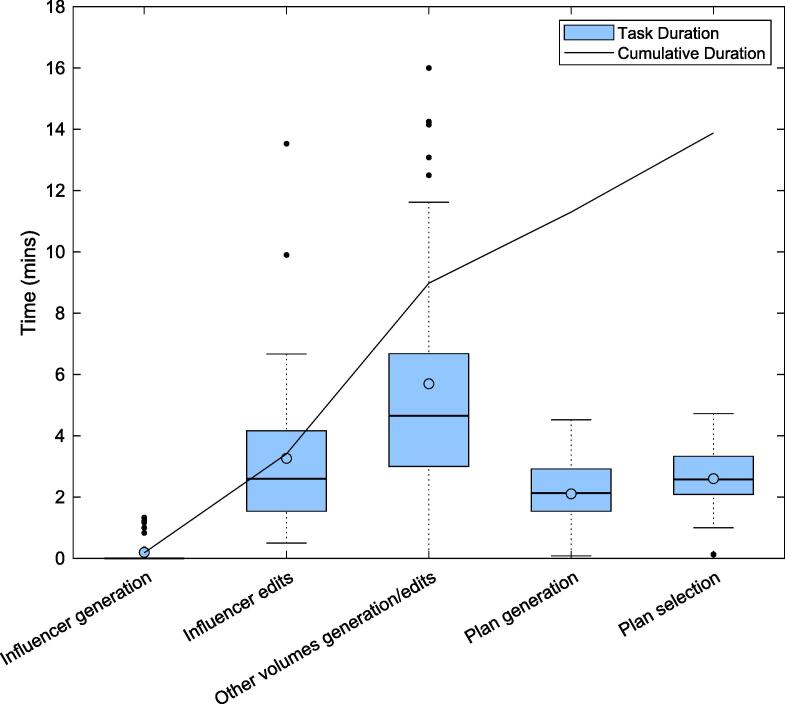
Duration of each discrete task identified in the kV CBCT-guided adaptive workflow for clinical patient fractions where the line denotes the median, and the circle denotes the mean duration of each step. The mean cumulative time of each task is overlaid.

The median duration of the adaptive planning process was 13.2 mins (7.7–32.5 mins), illustrated in Fig. 6. Tasks with the longest duration were ‘other volumes generation and edits’, followed by ‘influencer edits’.

## 4 Discussion

We have demonstrated that simulation-free radiotherapy with CBCT-guided online adaption is feasible in a real-world setting, minimising patient visits whilst accounting for anatomical changes associated with advanced cancer. The sCT generation accuracy was found to be adequate using our scoring tool, most unsupervised contours agreed with corresponding supervised contours reporting a DSC > 0.85, adaptive plan target coverage outperformed scheduled for all fractions and the oART process duration took a median of 13.2 mins.

This study adds to experience reported in the few existing publications evaluating simulation-free CBCT-guided oART [20], [21]. Previous studies focused on bony metastases treated with single fraction therapy. Although a limited number of patients were investigated in the present study, the anatomic site and physiology of disease varied, as detailed in Table S1. The evaluations presented extend those in the existing literature to consider a complex and varied palliation case mix including soft tissue targets and multiple dose regimens, including SIB.

The ‘body’ and ‘bones’ visual, scoring system was developed specifically for the CBCT-guided oART system, to act as a decision aid during adapted fractions to identify unrepresentative synthetic CTs prior to treatment delivery. The system was found to perform satisfactorily for simulation-free applications using this tool. When using the simulation-free pathway large changes are anticipated between the reference (i.e. dCT) and treatment images, and DIR is more likely to contain deformation artefacts when large geometric differences are seen [23], [24]. It is of great importance to identify and assess these artefacts prior to palliative plan delivery, as many treatments are delivered in a single fraction.

A synthetic CT containing voxels with incorrect HU values will result in inaccurate dose calculation and misrepresentation of dose metrics used for clinical decision making. This is driven by the AXB algorithm reporting dose to medium, where categorisation of voxels as bone results in a lower dose compared to soft tissue [25]. In instances where the ‘bones’ contour was larger than session CBCT bony anatomy, AXB would interpret affected voxels as bone erroneously, and would increase the dose to the affected region to achieve the required DVH metrics in the adapted plan. The actual dose delivered to the tissue represented by impacted voxels could be up to 5% higher per cm of expansion, than displayed.

Previous studies [20] have only evaluated the synthetic CT post-treatment. Without an online synthetic CT evaluation, there is a risk of delivering dose different to displayed as the synthetic CT is not visible, making such errors difficult to detect. Practically, it was observed that variation in pelvic tilt, patient weight and ventilation state of the dCT compared to the session CBCT, increased the likelihood of erroneous surrogate contours, while careful reproduction of the dCT patient position improved synthetic CT accuracy.

Comparison of the unsupervised and supervised contours demonstrated that auto-segmentation of palliative contours was generally well executed by the oART system in line with a CBCT-guided oART investigation of head and neck structures [26]. However, the broad range of individual DSCs reported across different fractions, visualised in Fig. 3, highlights expert supervision of contours is required. Furthermore, time required to correct impacted contours may present a barrier to widespread oART implementation as palliative patients are typically predisposed to gas due to low activity levels, negatively impacting CBCT image quality. The comparison of stomach contours stratified by image quality demonstrated a difference in median DSC, suggesting poor image quality may help predict poor unsupervised contour accuracy.

Other studies [16], [17], [19], [20], [21] qualified the number of slices edited in clinical online adaptive sessions, rather than contour overlap. The use of DSC quantifies the system’s contour generation accuracy and completing the assessment offline enabled the supervised arm to be as objective as possible. If the ground truth was defined during an online adaptive session, less accurate contours may have been accepted if not perceived to impact the adaptive plan. The qualitative metric might correlate better with the duration of contour edits but would require further investigation.

CBCT-guided oART accounted for the anatomic changes encountered in the simulation-free approach, including patient setup and disease progression. The adapted plans consistently achieved better target coverage compared to the scheduled plan. However, the poor target coverage observed in scheduled plans may be attributed to the oART system auto-registration of the dCT and session CBCT. For this reason, the superior performance of oART plan generation as reported here, could be over-estimated. The comparison of adapted and scheduled OAR doses did not distinguish a consistent trend, which was attributed to target coverage being the primary plan objective. The results indicated OAR sparing was not degraded with adaption, however statistically supported conclusions were not possible due to the small OAR sample size. In order to determine the true impact of oART in the simulation-free palliative setting, studies comparing patient outcomes of oART and IGRT are needed.

The median duration of the adaptive process for clinical patients was 13.2 min, well within the identified interval of 20 min. This result was consistent with timing studies reported by other CBCT-guided adaptive users [16], [17] particularly a simulation-free palliative study [21], but longer than a standard palliative fraction. The measured adaptive process duration also aligned with another pathway, utilising diagnostic images and MRI-adaptive system [19].

Steps consuming the most time involved assessing or correcting auto-generated structures. This remains a barrier to widespread implementation of CBCT-guided adaption for simulation-free palliation, as patients may not tolerate extended time in the treatment position. The duration also increases the likelihood of intrafraction motion [27], which may degrade adaptive accuracy gains [28]. Furthermore, current local protocols require a clinician and physicist to attend each fraction, placing a substantial resource burden on a department.

This study helped identify three barriers to simulation-free CBCT-guided oART; 1) DIR generation of the synthetic CT, 2) intrafraction motion, and 3) poor image quality of the session CBCT. The synthetic CT may not accurately represent a patient with variable anatomy due to DIR limitations, which excluded mobile targets such as extremities. The oART system evaluated does not have integrated intrafraction motion monitoring, consequently, lung and upper gastrointestinal targets were not considered. Furthermore, current local practice required patients palliated for cervical spine and head and neck malignancies be simulated with a thermoplastic mask prior to treatment, removing eligibility for this study. The final and largest barrier for simulation-free oART was CBCT image quality. Very few abdominal, soft-tissue lesions could be adapted, even with a high likelihood of interfraction variation due to poor soft-tissue CBCT contrast. Experienced clinicians anecdotally struggled to differentiate target volumes from healthy tissue due to low-quality images. Additionally, the occurrence of obstructive artefacts from intestinal gas further degraded session images.

Advancement in CBCT quality may resolve these issues. Improved visualisation of patient anatomy could improve system auto-segmentation accuracy and enable adaption for previously ineligible patients. Furthermore, it could lead to direct CBCT dose calculation, removing the synthetic CT from the process.

In conclusion, the CBCT-guided online adaptive workflow has been successfully applied to a simulation-free, palliative cohort of patients. The high dose conformity of adaptive plans may improve palliative outcomes, including pain reduction or physiological functionality. Care should be taken with the technology as it relies heavily on a synthetic CT generated through DIR, and thus is sensitive to the limitations of that process. The use of this technology in the context of palliative radiotherapy represents a marked change in the way these treatments are typically delivered, which may lead to further utilization of radiotherapy for palliative patients.

## Declaration of Competing Interest

The authors declare the following financial interests/personal relationships which may be considered as potential competing interests: Collaborative research agreement with Varian Medical Systems that partially funded this study.
