# Supplementary material for: Evaluation of the clinical feasibility of cone-beam computed tomography guided online adaption for simulation-free palliative radiotherapy
Source: Phys Imaging Radiat Oncol. 2023 Aug 31;28:100490. doi: 10.1016/j.phro.2023.100490 (PMC10495619; doi:10.1016/j.phro.2023.100490)
Supplement: Supplementary data 1 [file mmc1.pdf]

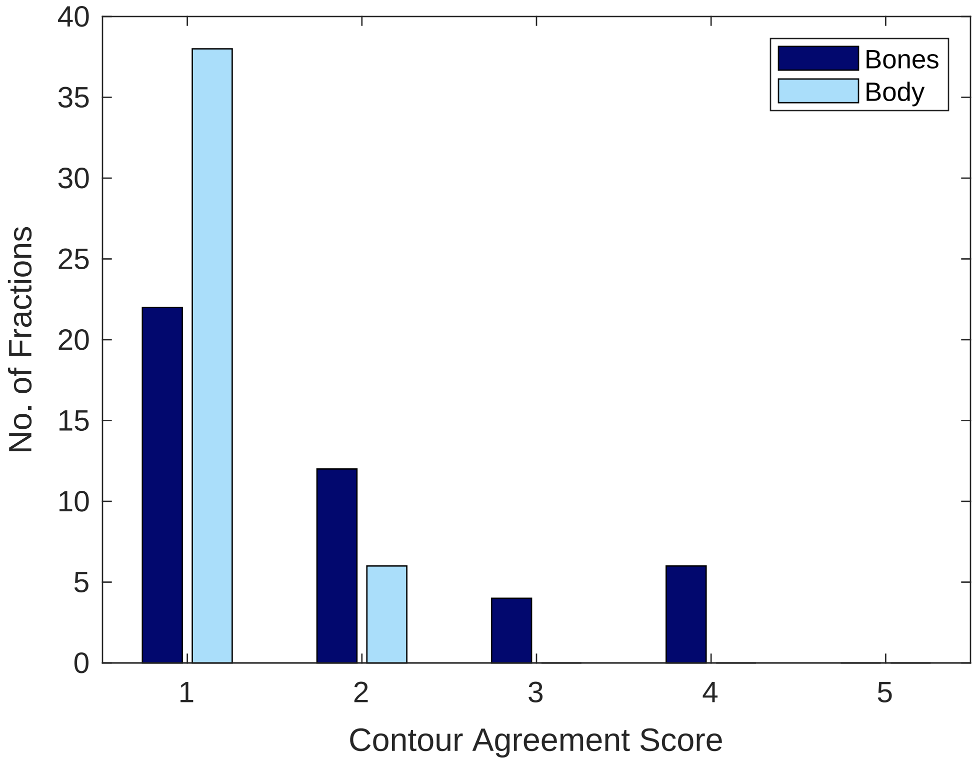

Figure S1. Histogram of qualitatively assigned registration scores per individual fraction. Each score describes the alignment of the 'body' contour to the session CBCT patient outline, and 'bones' contour to the session CBCT bone edges, acting as a surrogate of synthetic CT accuracy.
