# Supplementary material for: Evaluation of the clinical feasibility of cone-beam computed tomography guided online adaption for simulation-free palliative radiotherapy
Source: Phys Imaging Radiat Oncol. 2023 Aug 31;28:100490. doi: 10.1016/j.phro.2023.100490 (PMC10495619; doi:10.1016/j.phro.2023.100490)
Supplement: Supplementary data 2 [file mmc2.docx]

**Supplementary Materials**

| Table S1 Palliative cohort plan characteristics. Note: in the case of multiple dose levels, the highest dose prescribed was reported. | | | | | | |
| --- | --- | --- | --- | --- | --- | --- |
| Anatomic Site | | **No. of offline treatment sites** | | **No. of online adapted treatment sites** | |  |
| Thorax | | 4 | | 3 | |  |
| Thoracic Spine | | 0 | | 3 | |  |
| Abdomen | | 4 | | 1 | |  |
| Pelvis (bony) | | 1 | | 8 | |  |
| Pelvis (soft tissue) | | 0 | | 2 | |  |
| Lumbar Spine/Sacrum | | 1 | | 6 | |  |
| Dose Fractionation | | **No. of online adapted treatment plans** | | | |  |
| 8 Gy in 1 fx | | 4 | | | |  |
| 10 Gy in 1 fx | | 4 | | | |  |
| 14 Gy in 1 fx | | 8 | | | |  |
| 20 Gy in 4 fx | | 1 | | | |  |
| 25 Gy in 5 fx | | 2 | | | |  |
| 30 Gy in 5 fx | | 4 | | | |  |
|  | |  | |  | |  |
| Table S2 Scoring criteria used to visually assess the agreement between synthetic CT ‘body’ and ‘bones’ contours (surrogates of DIR accuracy) and the session CBCT anatomy | | | | |  |  |
| Score | **Phrase** | | **Description** | |  |  |
| 1 | Locally aligned | | Contour aligned within 2 mm of CBCT inside the VOI | |  |  |
| 2 | Mild misalignment | | Contour aligned within 2-5 mm of CBCT inside the VOI | |  |  |
| 3 | Moderate misalignment | | Contour aligned within 5-10 mm of CBCT inside the VOI | |  |  |
| 4 | Substantial misalignment | | Contour aligned within 10-20 mm of CBCT inside the VOI | |  |  |
| 5 | Alignment unacceptable | | Contour unable to be aligned within 20 mm of CBCT inside the VOI | |  |  |
